# Immediate Auditory Repetition of Words and Nonwords: An ERP Study of Lexical and Sublexical Processing

**DOI:** 10.1371/journal.pone.0091988

**Published:** 2014-03-18

**Authors:** Xiaorong Cheng, Graham Schafer, Patricia M. Riddell

**Affiliations:** 1 Key Laboratory of Adolescent Cyberpsychology and Behavior (CCNU), Ministry of Education, Wuhan, China; 2 School of Psychology, Central China Normal University, Wuhan, China; 3 School of Psychology and Clinical Language Sciences, University of Reading, Reading, United Kingdom; University of Leicester, United Kingdom

## Abstract

ERPs were elicited to (1) words, (2) pseudowords derived from these words, and (3) nonwords with no lexical neighbors, in a task involving listening to immediately repeated auditory stimuli. There was a significant early (P200) effect of phonotactic probability in the first auditory presentation, which discriminated words and pseudowords from nonwords; and a significant somewhat later (N400) effect of lexicality, which discriminated words from pseudowords and nonwords. There was no reliable effect of lexicality in the ERPs to the second auditory presentation. We conclude that early sublexical phonological processing differed according to phonotactic probability of the stimuli, and that lexically-based redintegration occurred for words but did not occur for pseudowords or nonwords. Thus, in online word recognition and immediate retrieval, phonological and/or sublexical processing plays a more important role than lexical level redintegration.

## Introduction

In an influential article, Norris, McQueen and Cutler [1] ask “Does information resulting from word (lexical) processing feed back to alter the immediate operation of prelexical processes [?]” (p.300). But before word-level representations can influence processing, they need to be learned. Here, we report an investigation concerning the relationship between word-likeness (lexicality), the immediate recognition of an auditory stimulus and repetition. The study was designed to determine how the lexicality of a stimulus affects both its immediate recognition and repeated processing.

The process of learning a word involves recognizing on subsequent encounters that it is no longer novel. The way in which existing word knowledge influences the neural processing of heard words is the subject of ongoing research [1], [2], and is of fundamental importance to the study of vocabulary acquisition [3], [4], [5].

Immediate auditory repetition is a useful method to ascertain the extent to which existing long-term lexical representations influence processing of heard words. Indeed, processes invoked by repetition have been argued to be directly related to those related to vocabulary learning [6]. Following auditory presentation of a known word, its mental representation remains active for some time, and this activity can be measured [7], [8]. If the heard word is then repeated some short time later, differential behavioral (e.g., priming) and neural responses to repeated and nonrepeated stimuli can be observed [9], [10], [11]. Short-term repetition priming is generally held to be caused by still-excited representations modulating the brain's response to the repeated stimulus [12], [13]. Such representations could in principle be lexical (i.e., represent the word in its entirety), or might be composed of a number of sublexical units, together making up that word's mental representation [14].

Redintegration refers to the process by which permanent representation in long-term memory modulates temporary representation in short-term memory [7]. In cases where repetition of a known word is involved, *and* where the level of representation is of words as entire units, such modulation is referred to as *lexical* redintegration [15], [16]. Lexical redintegration can in principle be influenced by any aspect of a word which is stored with it in the lexicon. Such processes would require prior access to the lexicon in order to operate.

The existence of whole-word level processes is often inferred from studies contrasting performance on words with nonwords. Words, it is argued, gain support from the lexicon in a way that nonwords cannot. Such models, emphasizing the representation of whole word redintegration, include those of Hulme and colleagues [17], who showed that serial recall is better for words than nonwords. Similar results have been reported by Jefferies, Frankish, and Lambon Ralph [18] and Ruchkin et al. [19]. Other important models of memory which rely on a word level representation include those of Brown, Preece, and Hulme [20], Burgess and Hitch [21], Lewandowsky and Farrell (2000), and Page and Norris (2008).

It is important to note that Schweickert (1993) in fact suggested that redintegrative processes can occur at either a lexical or a sublexical level. In distinction to lexical representations, *sublexical* representations do not require access to the lexicon before they can modulate neural responses to a word heard for a second time. Disentangling the role of word-level from sublexical representations in memory tasks is complicated by the fact that sublexical processes also function in the case of whole words—just as they do for word-like nonwords. The prime sublexical factor of interest here is phonotactic structure (i.e., the phoneme sequence of the items and their similarity to words in the language). It is argued that phonotactic variables (e.g., biphone frequency) influence reconstruction of traces in memory via long-term knowledge of the phonotactic properties of the language [22]–[24].

Sublexically-mediated effects have been shown in lexical decision tasks [25]. Further, it has been shown that performance for high phonotactic probability nonwords is better than performance for low phonotactic probability nonwords in serial recall and item recognition tasks [16], [22], [26].

Further complicating the distinction between lexical and sublexical representations, observations that appear as sublexical effects might actually be caused by the ability of word-like stimuli to activate lexical level representations. Thus, in a lexical decision task, nonwords with high phonotactic probabilities were responded to more slowly than nonwords with low phonotactic probabilities [27], [28]. According to Vitevitch and Luce [27], the inhibitory phonotactic probability effect in their lexical decision task was caused by greater competition among lexical neighbors activated by nonwords with higher phonotactic probabilities. According to this explanation, the effect of relative phonotactic probability is via *lexical* level redintegration. In support of such an interpretation, in a direct comparison of lexical and sublexical accounts of redintegrative effects for nonwords, Roodenrys and Hinton [26] compared the effects of phonotactics and number of word neighbors in a serial recall task. They reported that recall was influenced by the number of lexical neighbors of a nonword, rather than its phonotactic structure, and argued that this was good evidence for the lexical level as the sole locus of redintegration.

Although behavioral tasks such as lexical decision strongly suggest that sublexical processes mediate word-level tasks, and can be used to draw inferences about interactions between lexical and sublexical levels, they are relatively uninformative in respect of the online processes at work. In the present study, we compare the time course of the brain's response to recently-repeated examples of the *entire* word with its response to very similar stimuli which contain the root of that word, and nonwords, using event-related potentials (ERPs). The lexicality of our stimuli was carefully manipulated to allow us to measure word-level modulation of the response to such auditory repetition. The high temporal resolution of the event-related potential (ERP) method makes it ideal for the neurophysiological study of lexicality effects in lexical processing [29]–[32].

For maximal relevance to the role of processes relevant to both learning newly-encountered words, and auditory perception in general, and to be independent of reading, presentation in the auditory domain is required. Our study closely resembles that of Deacon et al. [12] described below, inasmuch as we manipulate lexicality of stimuli presented repeatedly to participants, but in the auditory, rather than the visual, domain (see [11] for comparison between domains).

A further important motivation for our use of ERPs is that the neural response to immediate repetition, and to word-like versus nonword-like stimuli are both well-documented, and distinct. In essence, the ERP response to a repeated versus a non-repeated stimulus starts before the ERP response associated with activation of a word-level representation [33]–[35]. ERPs to repeated stimuli also contain late components including effects of repetition at 250 ms, possibly the result of contact between perceptually-based and memory-based representations [36], [37]; whilst effects at 400 ms are generally associated with semantic or lexical access (but see [12], [38]), so that N400 modulation is seen for example in repetition of semantically-related words [39], [40]. A manipulation known to affect any repetition effect is the interval between the first presentation and the repetition [9], [41]. Immediate repetition appears stronger than delayed repetition [42] and so we use immediate repetition to maximize any possible repetition effects for all stimuli.

### ERP responses to lexicality

Numerous ERPs studies show differential responses to stimulus lexicality [12], [43]. Sublexical processes can be investigated by manipulation of phonotactic probability in stimuli which are nonwords. ERP responses modulated by such sublexical processes are apparent relatively early. Bonte and colleagues [44] studied ERP responses to phonotactic probabilities in an oddball paradigm. Differential ERP responses to auditorily-presented Dutch nonwords with high (*notsel*) versus low (*notkel*, *notfel*) phonotactic probabilities started about 160 ms from stimulus onset; further, the mismatch negativity (MMN) to the high phonotactic probability nonword (*notsel*) was significantly higher than the MMN to a low phonotactic probability nonword (*notkel*) (see also [45]).

Holcomb and Grainger's [34] study also associated the N250 with the sublexical-lexical interface. Intriguingly, Sereno, Rayner, and Posner [46] reported differences occurring within 100 ms post-onset between visually-presented words, pronounceable pseudowords, and unpronounceable consonant strings during a lexical decision task. Huber and colleagues [47] ascribed such early differences to visual processing of letters.

The N400 component is generated in both visual and auditory tasks, and is generally, though not exclusively, associated with semantic processing [43], [48], [49]. The N400 is sometimes argued to reflect semantic integration, i.e., post lexical processing [50], [51].

In Rugg and Nagy's (1987) study, legal nonwords showed a repetition effect in N400, whereas illegal nonwords did not. There are two alternative explanations for such repetition effects for legal nonwords: (1) a lexical account, in which legal nonwords have orthographic and phonological overlaps with real words, which enable them to activate associated real words [52]; (2) a sublexical account, in which repetition priming effects in the N400 component reflect orthographic or phonological priming in the absence of semantic processing [31]. Although N400 is widely regarded as indexing semantic analysis, not all data support this view. Deacon and her colleagues [12] conducted an ERP study which compared N400 repetition priming for both derived nonwords versus words, and for nonderived nonwords (unrelated to real words) versus words, presented visually. Derived nonwords were legal and derived by changing one or more letters in a real word, such as *tolip* derived from *tulip*; whereas nonderived nonwords were orthographically legal, but not easily linked to any real word, such as *loppir* and *quapt*. Analogous results for repetition priming of both classes of nonword strongly suggested that N400 effects in repetition priming are related to orthographic, or possibly phonological, analysis, rather than semantic analysis. Deacon et al. argued that their data, considered with that of Rugg and Nagy [32] suggest that the N400 repetition priming effect is caused by the legal status of the letter strings used in both studies, rather than activation of words in the lexicon, and that such effects occur independent of lexical access because “N400 and N400 semantic priming effects were obtained for legal nonwords derived from actual words in Experiment 1 and N400 and N400 repetition effects were also obtained for legal and nonderivational nonwords in Experiment 2” (Deacon, et al., 2004, p.68).

Deacon et al.'s (2004) arguments are consistent with the Merge model [1], which proposes that the processing of a word is in its early stages is no more than the sum of sublexical processes. Our study is a limited replication of Deacon et al.'s study, with careful manipulation of lexicalities, performed in the auditory rather than visual domain, and particularly concerned with processes involved when stimuli are repeated.

### Design of the study

In the present study, we presented auditory words and nonwords to participants twice in immediate succession. The first presentation elicits word recognition, while the second simulated a retrieval stage in which lexicality (or otherwise) could play a part (or not) in reconstruction of the representation of the just-heard stimulus. To investigate lexical and sublexical processes, we manipulated the phonotactic probabilities of the nonwords in our study. Nonwords with high phonotactic probabilities were derived from real words; we term these *pseudowords*. Nonwords with low phonotactic probabilities were chosen to have no real word neighbors; we term these *nonwords*. Pseudowords presumably license lexical level auditory redintegration via their word roots. Nonwords on the other hand do not license lexical level redintegration. Participants were exposed both to a word, and to a pseudoword with that word as its root, in different blocks of the experiment.

The task we set our participants was designed to encourage them to pay attention to the phonological form of the stimuli, whilst allowing them to take advantage of the lexical status of any real words they heard. Importantly, the task could not be solved by assessing whether or not stimuli were real words.

According to *lexical* redintegration models, effects of repetition should differ between pseudowords and nonwords because only the former are subject to lexically-mediated processes. On the other hand, *sublexical* theories predict an equivalence between these stimuli: both are composed of legal fragments. If there is any difference between ERPs elicited to pseudowords and nonwords due to lexical redintegration, they should occur at or after N400 because N400 indexes lexical or post lexical processing.

## Methods

### Ethics statement

This study was approved by the ethics committee of the University of Reading. Prior to testing, written informed consent was obtained from all individuals for data collection, use, and publication.

### Participants

Thirty-six right-handed native British English speakers participated. Data from 12 participants were rejected (9: too many artefacts in the EEG recording i.e., fewer than 80% good trials; 2: extraneous 25 Hz noise in EEG recording; 1: over 10% (16%) overall error rate in the behavioral task). The remaining 24 (five male) participants had mean age 20.2 years (18–28, SD = 2.17). Nine were paid, recruited opportunistically from the campus of University of Reading, the remainder were Psychology undergraduates, rewarded by course credit.

### Stimuli

Words were selected using the MRC Psycholinguistic Database [53]. Because stimuli were to be used in a further study with 5–6 year old children, the age of acquisition of individual words was set to be 6 years or lower; 340 such words were obtained. To minimize word frequency effects, words with Kucera-Francis written frequency >152 were excluded [54]. Compound words (e.g., sunshine) were also excluded. The remaining 169 words were converted to pseudowords by changing a single phoneme, for example, *game* to *gome*. The position of the changed phoneme in any given word was not controlled.

One hundred and twenty nonwords were chosen at random from the ARC online nonword database [55]. Nonwords were chosen to be phonologically legal, but without phonological neighbors. Only one nonword was kept from groups of homophones.

Auditory stimuli, spoken by a middle-aged male native British English speaker, were recorded at 22050 Hz (16 bit mono) using Goldwave software. Recordings were filtered for noise reduction, and stimulus volume was normalized by equating pressure peak amplitudes.

#### Pretest

For our purposes, it was important that pseudowords and nonwords had genuinely different status (the former with a real word root; the latter as far as possible unrelated to any word in the participant's lexicon). However, a mispronounced or degraded word can be perceived as the word itself [56], [57]. We therefore ran a pretest to ensure that (a) pseudowords were not misperceived as their word roots or phonological neighbors; (b) nonwords were genuinely distinct from real words. Five participants (mean age  = 21.2 years; 18–28, 4 female) heard the 169 pseudowords and 120 nonwords in random order through Plantronics PC Headset Binaural NC Multimedia headphones. Participants were instructed that, after each item, they were to say as many words as they could which sounded like the item they had just heard. Participants had 10 s to respond. Verbal responses were recorded for subsequent transcription.

All verbal responses were transcribed by the first author, with assistance from helpers who did not know the aim of the pretest and had not heard the cue word. If more than three participants generated the same word, and less than 4 other words in total for an item, it was considered “bad” and deleted from the stimulus list. By this method, 122 pseudowords and 113 nonwords were retained as good stimuli^1^.

#### Final stimulus list

For the final stimulus list, 50 nonwords were chosen randomly from all good nonwords. Fifty words and their corresponding pseudowords were chosen from all good pseudowords. All 150 stimuli were monosyllables and they did not reliably differ in recording length and number of phonemes (see Table 1 and Supporting Information Table S1: Words, Pseudowords and Nonwords used in the study).

**Table 1:** Attributes of stimuli.

	Words (SD)	Pseudowords (SD)	Nonwords (SD)	p^d^ (if applicable)
**AoA** a	250 (32.7)	N/A	N/A	N/A
**K-F frequency** b	37.4 (36.1)	N/A	N/A	N/A
**W-S frequency** c	62.5 (58.4)	N/A	N/A	N/A
**Number of phonemes**	3.42 (0.50)	3.42 (0.50)	3.60 (0.50)	.116
**Recording length (ms)**	551 (71.6)	548 (76.4)	547 (59.4)	.954
**PPoFP**	0.057 (0.031)	0.046 (0.032)	0.026 (0.032)	<.001
**PPoFB**	0.005 (0.005)	0.004 (0.005)	0.001 (0.002)	<.001
**PPoSP**	0.16 (0.05)	0.14 (0.05)	0.10 (0.04)	<.001
**PPoSB**	0.008 (0.007)	0.007 (0.007)	0.003 (0.002)	<.001

*Note*. N/A =  Not Applicable.

a AoA was multiplied by 100 in MRC database.

b K-F frequency  =  Kucera-Francis written frequency.

c W-S frequency  =  Word frequencies in written and spoken English [78]. Not all words appeared in this corpus, so W-S frequency in this table was only from those words existing in the corpus. If a word has two or more written and spoken frequencies in the corpus because it is a noun and also a verb or has two meanings, its word frequency in this study is the total of all frequencies. Eight words in stimuli were not included in the W-S frequency corpus.

d p value from ANOVAs for attributes between three lexicalities.

Four positional-specific phonotactic probabilities were calculated for each stimulus from computer-readable IPA transcriptions, using an online phonotactic probability calculator [58]. These probabilities were: (1) phonotactic probability of the first phoneme (PPoFP), defined as the phonotactic probability of the first phoneme at the first position in a word, (2) phonotactic probability of the first biphone (PPoFB), defined as the phonotactic probability of the first biphone, i.e., the first phoneme in the first position and the second phoneme in the second position, (3) the sum of phonotactic probabilities of positional segment (PPoSP), defined as the sum of phonotactic probabilities (PP) of each phoneme in a specific position, and (4) the sum of phonotactic probability of position-specific biphones (PPoSB), defined as the sum of phonotactic probabilities of each biphone in a specific position. All individual PPs are log (base 10) values.

The four PPs differed between three lexicalities (ps<.001). In paired comparisons, PPoFPs and PPoFBs of nonwords were lower than those of pseudowords (p = .001 and p = .006 respectively) and words (both ps<.001); PPoSPs and PPoSBs of nonwords were lower than those of pseudowords (ps<.001) and words (ps<.001) (see Table 1 for attributes of stimuli).

#### Trial construction

To obviate priming between words and their corresponding pseudowords, stimuli were presented in two blocks (25 items in each condition per block); words and corresponding pseudowords were not presented in the same block. All participants heard two blocks of stimuli and the presentation order of the blocks was counterbalanced across participants.

There were three items per trial. Participants were instructed to judge whether all three items in each trial were the same. Experimental stimuli were presented twice, as the first and second items in the trial. All experiment trials took the form A+A+A or A+A+B. Fifteen out of the 50 trials for each lexicality were followed by a different third item. Therefore, in one block, 17 out of 25 trials in each of the three lexicality conditions were allocated to A+A+A, and 8 to A+A+B, i.e., 51 “Yes” responses and 24 “No” responses across the three lexicalities. In the other block, 18 were allocated to A+A+A and 7 to A+A+B, i.e., 54 “Yes” responses and 21 “No” responses.

Sixty filler trials with differing first and second items (all therefore “No” responses) were introduced to make the repetition paradigm unpredictable. Of the filler trials, 20 took the form A+B+C, 20 A+B+A, and 20 A+B+B. All items in filler trials were randomly chosen (without replacement) from previously-recorded stimuli from a previous study with similar selection criteria to the present study. Words, pseudowords and nonwords were evenly distributed in the filler trials, and items for each lexicality group were evenly placed in the first position in the three combinations of filler trials.

Further, in filler trials which had a word or a pseudoword in the first or second position, half of the words were the wordroot of the following or preceding pseudowords. With these kinds of filler trials in the experiment, participants could not perform the task by using solely the identity of the first phoneme (because the first phoneme in some words and their corresponding pseudowords were the same). This manipulation encouraged participants to listen to all information of the first two items of each trial.

The three combinations of filler trials were allocated approximately equally to the two blocks, giving 51 “Yes” and 54 “No” trials in one block, and 54 “Yes” and 51 “No” in the other. The total numbers of “Yes” and “No” responses were thus equal across the experiment.

### Procedure

All stimuli were played through a Sony SRS ZP1000 - PC multimedia speaker at a comfortable listening level. Participants initiated each trial by pressing the space bar, and could take a break whenever they chose.

At the start of each trial, a central black fixation cross was then presented on the monitor for 700 ms, after which time the cross changed color to red for a further 300 ms, indicating imminent presentation of the trial. The red cross was followed by a black box of dimensions 60 mm W×30 mm H, the purpose of which was to restrict participants' eye movements. Visual angles were modulated to be less than 6.8 degrees horizontally and 3.4 degrees vertically by keeping the distance between a participant and the screen over 50 cm. (Participants had previously been told to avoid blinks or other movements when the black box appeared.) Auditory presentation of the first item in the trial commenced 500 ms after appearance of the black box, which stayed on the screen for a further 1800 ms. After offset of the box, the above procedure from black cross onset to black box offset was repeated for the second auditory item. After the offset of second presentation of the black box, the third item in a trial was played and a question mark presented on the screen. Participants were instructed to respond “Yes” with left hand or “No” with right hand on a response box according to whether the three items in the trial had been the same.

Before the recording phase started, the procedure was demonstrated and participants were trained not to move or blink during presentation of the black box. The ERP recording lasted about 35 minutes (i.e., approx 20 mins presentation, plus responses and breaks).

### EEG recording and data analysis

Electrophysiological (EEG) signals were collected from the scalp with an Electrical Geodesics GSN 200 sensor net system with 128 channels (Electrical Geodesics, Inc., Eugene, OR), amplified by the EGI NetAmps 200 amplifier with a bandpass of 0.1–100 Hz, and digitized at 250 Hz. The threshold for impedance was set at 50 kΩ and all sites were recorded with a vertex reference.

Electrophysiological signals were filtered with a 40 Hz low-pass filter. The EEG sessions were then segmented with a time window from 100 ms before stimulus onset to 1500 ms after it. Trials were labeled bad (a) in the case of eye movements (EOG over 70 μV) or (b) >10 bad channels (average amplitude >200 μV or transit amplitude >100 μV). Such trials were individually discarded. Participants' datasets were discarded altogether if more than 25% of trials were bad in any condition. In the final data analyses, the overall mean good trial rate was 95.1% (see Results). Single bad channels were replaced by the interpolation using a spherical spline algorithm [59]. After bad channel replacement, all segments for each condition of each participant were averaged individually. A polar average reference effect (PARE)-corrected reference was used [60], computed from the average of the entire surface of the scalp. Finally, ERPs were baseline corrected according to the recording of 100 ms pre-stimulus interval.

### Data analyses

Initially, we compared ERPs from the two presentations to establish that our experiment replicated common findings in immediate repetition of auditory stimuli, and that the ERP components we expected to observe, based on previous findings, were indeed present. Data analyses were then undertaken for the two presentations separately to investigate whether ERPs to the three lexicalities differed from each other in respect of specific individual ERP components identified a priori.

**Figure 1:**
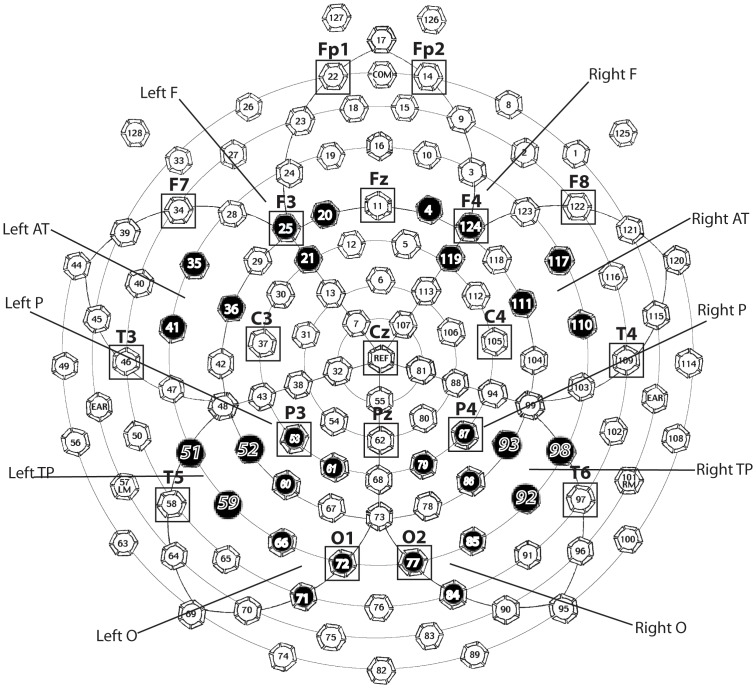
Chosen sensor layout for each cluster and approximate corresponding location of 10–20 system. Black sensors were chosen sensors in data analysis and sensors with rectangles show approximate correspondences of 10–20 system as chosen in previous. *Note*: F = Frontal, AT = Anterotemporal, TP = temporoparietal, P = Parietal, O = Occipital. These abbreviations are also used in all later figures and tables.

Three clusters on each hemisphere were chosen according to traditional 10–20 recording system on the net, i.e., frontal (F7, F8), parietal (P3, P4) and occipital (O1, O2). Two additional clusters, anterotemporal (Broca's area) and temporoparietal (Wernicke's area) on each hemisphere, were assessed as areas sensitive to spoken language processing [43]. This approach gave rise to five clusters of interest on each hemisphere, viz. frontal, anterotemporal, temporoparietal, parietal and occipital. Three sensors were chosen for each cluster, see Figure 1.

To investigate repetition effects, a 2×3×2×5 repeated measures ANOVA with the factors: Presentation (first and second), Lexicality (words, pseudowords and nonwords), Hemisphere (left and right) and Cluster (frontal, anterotemporal, temporoparietal, parietal and occipital) was conducted and effects involving Presentation reported. To further investigate the lexical and sublexical effects separately in each presentation, results involving Lexicality from the initial ANOVA were submitted to additional three-way ANOVAs (i.e., without Presentation as a factor). Simple main effects of Lexicality were investigated using additional ANOVAs as appropriate.

All ANOVAs results were adjusted with Greenhouse-Geisser adjustment where Mauchly tests of sphericity were significant (p<.05). P values of all multiple comparisons for Lexicality were adjusted by Bonferroni correction.

## Results

### Accuracy during the ERP task

Accuracy of responses for each lexicality in the behavioral task differed by condition, F (2, 46) = 3.29, p = .046. However, paired comparisons did not show any significant difference between lexicalities, ps>.07.

### ERP results

**Figure 2:**
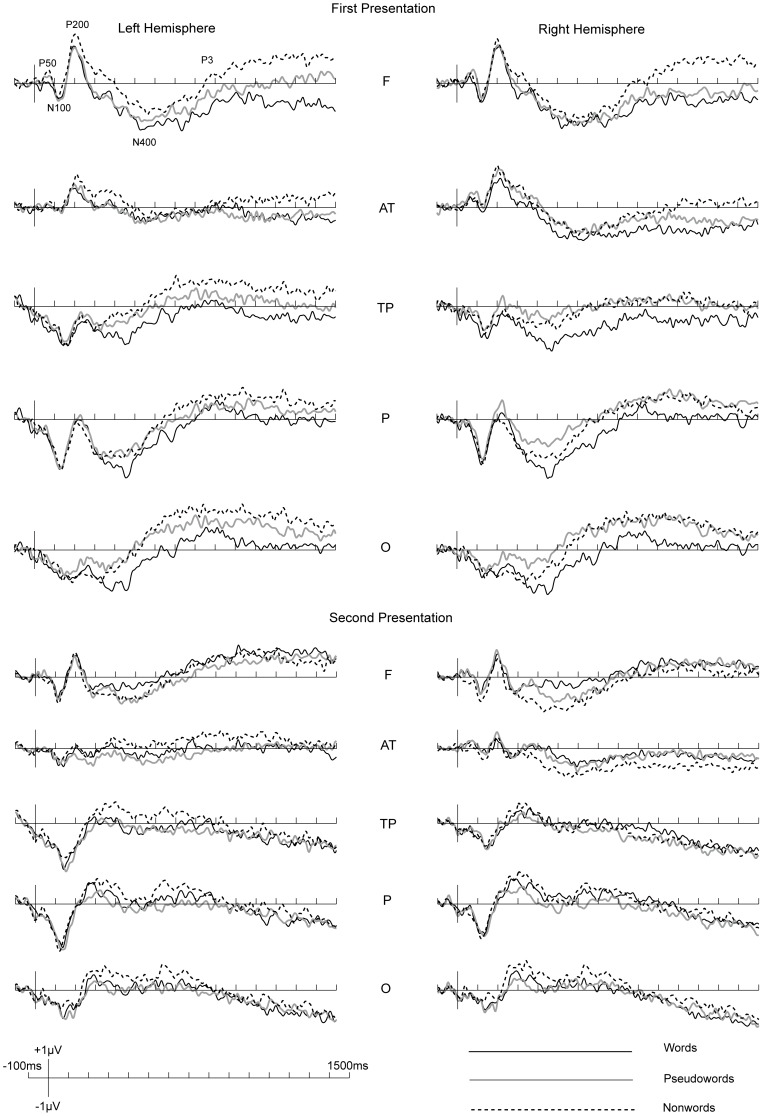
ERPs to all stimuli in two presentations.

ERP waveforms to the three lexicalities in the two presentations are shown in Figure 2. Inspection of the figure suggests that ERP waveforms in the present study are very similar to those reported in previous ERP studies on auditory word processing [43,e.g., 61]. Sequentially, P50, N100, P200, N400 and a late positive component, P3, were observed from the onset of stimuli.

**Figure 3:**
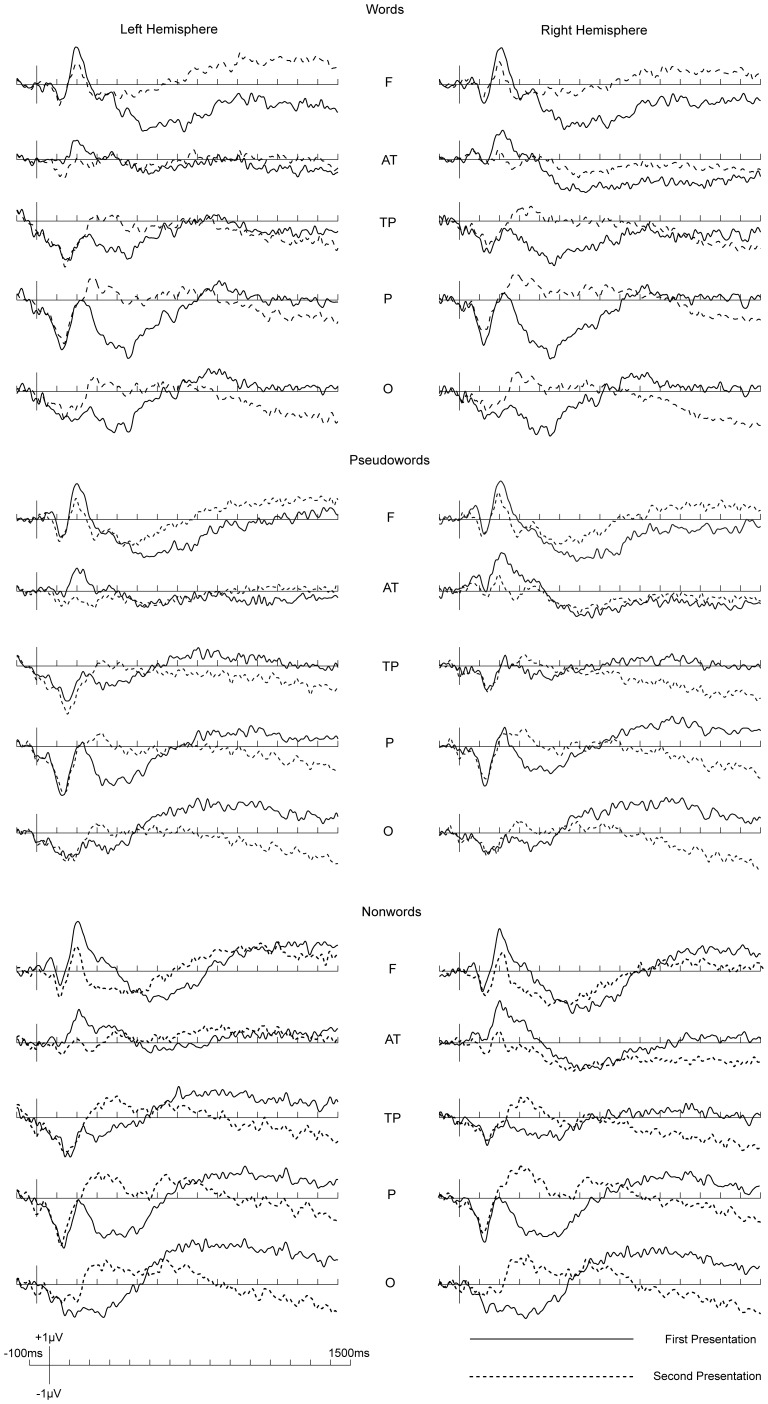
Direct comparisons of ERPs to all stimuli in two presentations.

Direct comparisons between the first presentation and the second presentation for words, pseudowords and nonwords respectively are shown in Figure 3, where it can be observed that divergence between the first presentation and second presentations started around P200.

Data analyses were conducted for mean amplitudes of P200 (200–300 ms), N400 (400–600 ms), P3 (700–1000 ms) and ERP in a very late time window (1200–1500 ms) because of visible divergence between the two presentations.

#### Comparisons of ERPs between presentations

In P200, the 2×3×2×5 ANOVA revealed a Presentation × Cluster interaction, F (2.05, 47.2) = 23.1, p<.001, and a Presentation × Lexicality × Cluster interaction, F (2.97, 68.4) = 3.69, p = .016. Further ANOVAs for each cluster revealed that in frontal and anterotemporal clusters, the mean amplitude of P200 in the first presentation was significantly more positive than that in the second presentation, F (1, 23) = 9.49, p = .005 and F (1, 23) = 34.7, p<.001 respectively; in occipital clusters, the mean amplitude of P200 in the first presentation was less positive than that in the second presentation, F (1, 23) = 12.8, p = .002.

In N400, the main effect of Presentation was significant, F (1, 23) = 12.0, p = .002. Two two-way interactions involving Presentation were significant, Presentation × Lexicality, F (1.93, 44.4) = 4.65, p = .016, and Presentation × Cluster, F (1.62, 37.3) = 4.39, p = .026. In temporoparietal, parietal and occipital clusters, the mean amplitude of N400 in the first presentation was significantly more negative than that in the second presentation, F (1, 23) = 9.91, p = .004, F (1, 23) = 39.0, p<.001 and F (1, 23) = 11.5, p = .003, respectively. Further, in temporoparietal clusters, two two-way interactions were significant, Presentation × Lexicality, F (1.94, 44.6) = 4.29, p = .021, and Lexicality × Hemisphere, F (1.86, 42.7) = 3.30, p = .050. In the left temporoparietal cluster, the simple main effect of Presentation was marginally significant, F (1, 23) = 3.30, p = .082. In the right temporoparietal cluster, the simple main effect of Presentation was significant, F (1, 23) = 13.2, p = .001 and the interaction between Presentation and Lexicality was significant, F (1.98, 45.6) = 5.03, p = .011. Further, to investigate if repetition effects for different stimuli differed from one another, mean amplitude of difference waves in N400s for the three lexicalities in right temporoparietal cluster were extracted and a one-way ANOVA with Lexicality was conducted. The simple main effect of lexicality was significant, F (1.98, 45.6) = 5.03, p = .011. In paired comparisons between all three levels of Lexicality, mean amplitude of N400s difference waves to words (−2.02 μV) was significantly larger than to pseudowords (−0.40 μV), p = .031, and nonwords (−0.62 μV), p = .049, but mean amplitude of N400s difference waves to pseudowords did not differ from nonwords, p∼1. Further one-sample tests revealed that only the difference wave of N400 to words was significantly different from 0, t(23) = −5.0, p<.001. In parietal clusters, two two-way interactions were significant, Presentation × Lexicality, F (1.90, 43.8) = 4.61, p = .017 and Lexicality × Hemisphere, F (1.86, 42.8) = 3.92, p = .030. In the right parietal cluster, the simple main effect of Presentation was significant, F (1, 23) = 40.7, p<.001, as was the Presentation × Lexicality interaction, F (1.89, 43.4) = 5.49, p = .008. Further, to investigate whether repetition effects for stimuli differed by Lexicality, mean amplitude of difference waves in N400s for the three lexicalities in the right parietal cluster were extracted and a one-way ANOVA with Lexicality was conducted. The simple main effect of Lexicality was significant, F (1.89, 43.4) = 5.49, p = .008. In paired comparisons between three levels of lexicality factor, mean amplitude of N400s difference waves to words (−2.60 μV) were significantly larger than to pseudowords (−0.91 μV), p = .015, but did not differ from nonwords (−1.90 μV), p = .628; mean amplitude of N400s difference waves to pseudowords did not differ from nonwords, p = .108. Further one-sample t-tests revealed that difference waves of N400 to words and nonwords were significantly different from 0, t(23) = −6.70, p<.001 and t(23) = −4.83, p<.001 respectively and the difference waves of N400 to pseudowords were marginally different from 0, t(23) = −2.05, p = .052.

In the late time window, two two-way interactions concerning Presentation were significant, Presentation × Lexicality, F (2, 46) = 3.68, p = .033 and Presentation × Cluster, F (1.71, 39.3) = 7.07, p = .004. In parietal and occipital clusters, the mean amplitude of ERP in the first presentation was significantly more positive than that in the second presentation, F (1, 23) = 5.12, p = .033 and F (1, 23) = 14.81, p = .001 respectively.

#### Results of mean amplitudes of ERPs in the first presentation

Significant results concerning Lexicality from the 3-way ANOVAs for each component and further significant effects in the first presentation were presented in Table 2.

**Table 2:** Significant output involving Lexicality in 3-way ANOVAs and further effects for mean amplitudes of ERP components in the first presentation.

Component	Effect	df	F	p	Further effect
**P200**	L×C	3.39, 78.0	3.49	.016	L×H in F, L in Left F: (W≈PW) < NW
**N400**	L	1.92, 44.0	4.78	.014	L×H, Left H: sig L, W<(PW≈NW)
	L×H	1.65, 38.0	3.87	.037	
	L×C	3.33, 76.6	2.18	.090	
**P3**	L	1.81, 41.7	4.63	.018	Sig L: W<NW
**1200–1500 ms**	L	1.80, 41.4	5.26	.011	Sig L: W<NW

*Note*: L =  Lexicality, C =  Cluster, H = Hemisphere, W = Words, PW = Pseudowords, NW = Nonwords, Sig = Significant.

#### P200 Component

**Figure 4:**
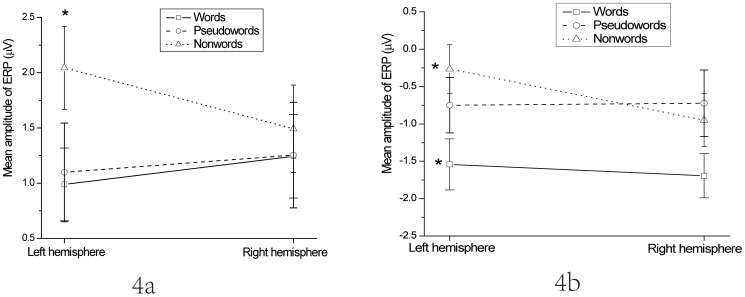
Interactions between Lexicality and Hemisphere. 4a, Interaction between Lexicality and Hemisphere in P200s in the first presentation in frontal clusters. 4b, Interaction between Lexicality and Hemisphere across all clusters in N400s in the first presentation. *Note*: * indicates <.05 significance level. Errors bars represent one standard error.

In P200, in frontal clusters, the Lexicality × Hemisphere interaction was significant, F(1.91, 43.8) = 4.51, p = .018 (see Figure 4a). In the left frontal cluster, the one-way ANOVA showed a significant simple main effect of Lexicality, F(1.85, 42.3) = 4.67, p = .017. In paired comparisons between the three levels of Lexicality, the mean amplitude of P200s to words (0.99 μV) did not differ from that to pseudowords (1.10 μV), p≈1; the mean amplitude of P200s to words was significantly less positive than the mean amplitude to nonwords (2.05 μV), p = .019; and mean amplitude P200 to pseudowords was also significantly less positive than that to nonwords, p = .024. In the right frontal cluster, the simple main effect of Lexicality was not significant, p>.50.

In anterotemporal clusters, only the main effect of Lexicality was significant, F(1.58, 36.3) = 3.88, p = .039. In paired comparisons, mean amplitude of P200 to words (0.82 μV) was marginally less positive than to pseudowords (1.14 μV), p = .097, but was significantly less positive than to nonwords (1.57 μV), p = .023; mean amplitude of P200 to pseudowords did not differ from that to nonwords, p = .167.

#### N400 Component

To further investigate the Lexicality × Hemisphere interaction in N400 (see Figure 4b), a two-way ANOVA with Lexicality and Cluster was conducted for each hemisphere.

In the left hemisphere only, the simple main effect of Lexicality was significant, F(1.67, 38.4) = 6.72, p = .005. In paired comparisons, the mean amplitude of N400 to words (−1.54 μV) was significantly more negative than to pseudowords (−0.75 μV), p = .018, and significantly more negative than to nonwords (−0.27 μV), p = .006. The mean amplitudes of N400 to pseudowords and nonwords did not differ, p = .132.

#### P3 Component

Paired comparisons for the main effect of Lexicality in P3 showed that the mean amplitude of P3 to words (−0.46 μV) did not differ from pseudowords (0.20 μV), p = .117, but was significantly less positive than for nonwords (0.69 μV), p = .011; mean amplitude of P3 to pseudowords did not differ from nonwords, p = .127.

#### Late time window

In the time window 1200–1500 ms after stimuli onset, in paired comparisons between the three levels of Lexicality, mean amplitude of ERP in the late time window to words (−0.56 μV) did not differ from pseudowords (0.18 μV), p = .176, but was significantly less positive than nonwords (0.94 μV), p = .002, and mean amplitude of P3 to pseudowords did not differ from nonwords, p = .075.

#### Results of mean amplitudes of ERPs in the second presentation

In the second presentation, significant results were only found in the N400. The two-way Lexicality × Hemisphere interaction was significant, F(1.72, 39.5) = 3.65, p = .041.In neither hemisphere were the simple main effect of Lexicality, or interactions involving it, significant, ps>.06.

#### Negative results: A Bayesian analysis

In the second presentation, we failed to find any statistically significant phonotactic probability effects in P200. This negative finding is noteworthy, and wishing as we do to draw conclusions about sublexical processing from this non-significant (‘negative’) result, a Bayesian analysis is indicated [62]. Calculation of a Bayes Factor allows an inference of ‘no effect’ to be distinguished from one of ‘no evidence of an effect’. The Bayes Factor (BF) directly calculated the ratio between the probability of the null hypothesis and the probability of the alternative hypothesis based on the observed data and BF over 3 can be considered as some evidence for the null hypothesis [63], [64].

The phonotactic probability effect in P200 in the first presentation was in the left frontal cluster, so if it showed in the second presentation, it follows that it should be observed again in P200 in the left frontal cluster. The phonotactic probability effect is best estimated by the difference between pseudowords and nonwords; thus we compared the mean amplitude of P200 for pseudowords and the mean amplitude of P200 for nonwords in the second presentation using a paired sample t-test, t(23) = 0.318, p = .754. We used the web-based Bayes Factor Calculator developed by Morey and Rouder [63], [64]. With the default scale r on effect size  = 1, JZS Bayes Factor  = 6.07, strongly suggesting a genuinely ‘null’ result for this effect.

## Discussion

In the present study, we recorded ERPs whiles participants listened to a word, a pseudoword, or a nonword presented twice in immediate succession. We observed significant repetition effects across all stimuli in P200s and the very late time window (1200–1500 ms post stimuli onset) across all clusters. The repetition effects in N400s across all stimuli were significant in the three posterior clusters. The general repetition effects were consistent with previous research which found that phonologically legal stimuli show repetition effects as early as P200 [11], [51].

In the first presentation, phonotactic probability effects were apparent in P200s over the left frontal cluster: P200s to words and pseudowords were significantly less positive than P200s to nonwords; lexicality effects were also found in N400s over the left hemisphere: N400s to words were significantly more negative than N400s to pseudowords and nonwords^2^. In the second presentation, there were no significant early differences between mean amplitudes of ERPs to the three lexicalities.

### Phonotactic probability effects in P200 in the first presentation

Our results do not support lexical redintegration models. According to such models, phonotactic probability effects are caused by lexically-mediated redintegration and as such will benefit pseudowords over nonwords, and will do this after lexical access. (Recall that nonwords in the present study did not have word neighbors.) Because N400 is argued to index lexical access [12], [38] or post-lexical access [49], [50], [51], in the initial presentation, only differences between pseudowords and nonwords which occur after the N400, support such models. Rather, our results in the first presentation suggest that phonotactic probability effects may have been caused by early phonological processing of different phonotactic probabilities, which distinguished nonwords both from words and from pseudowords. This conclusion has been recently confirmed by MacGregor, Pulvermuller, van Casteren and Shtyrov [65]. In an MEG study, MacGregor et al. found that differences in MEG responses between acoustic CVC words and pseudowords (which only differed from their corresponding words in the final phoneme) occurred at around 50–80 ms following the word recognition point. The word recognition point had been established to be around 300 ms in a behavioral gating task which participants completed before the MEG recording. Thus lexical processing that distinguished words from pseudowords started from around 350 ms after stimulus onset. Therefore, the phonotactic probability effects which emerged at P200 in our results can be attributed to phonological processing, rather than lexical redintegration.

Phonological processing skills arise, according to Metsala [66], from accumulating lexical knowledge, which drives phonological awareness and/or phonological sensitivity, i.e., skill in identifying and manipulating individual phonemes [67]. The relationship between lexical knowledge, phonological sensitivity and phonological processing has been discussed in terms of a lexical restructuring theory [66], [68]. According to this theory, increased phonological sensitivity will improve phonological processing ability. In turn, phonological processing can discriminate stimuli on the basis of lexicality, based on phonological probabilities. Thus, the early effect in P200, in which phonotactic probability determines differences in the initial presentation, is consistent with lexical restructuring theory.

The early phonotactic probability effect is also consistent with the word recognition model, Merge [1]. In Merge, prelexical processing continuously provides information for lexical processing to activate a set of lexical candidates in a bottom-up fashion; meanwhile prelexical processing itself determines which phonemes are in the input. Our results in the first presentation support this independent phonological processing view.

The early phonotactic probability effect in P200 in auditory word recognition is similar to the early syllable frequency effect found in a visual lexical decision task in Spanish [69]: P200 and N400s to words with high syllable frequency were more negative than N400s to words with low syllable frequency. The early modulation of P200 by syllable frequency has been confirmed in other ERP studies using visual word recognition [70], [71] and in a study of auditory phonotactic probability effects which measured MMN in typical developing children [72]. However, our results are less consistent with a study by Rossi and colleagues [73], who used a passive listening paradigm to investigate auditory phonotactic probability effects between phonologically legal and illegal (but pronounceable) pseudowords in German. They did not find an early phonotactic probability effect in P200, instead reporting a phonotactic probability effect in N400 for midline electrode sites only. Given that Rossi et al.'s study involved purely passive listening, it is possible that participants did not pay full attention to the early phonotactic difference. In contrast, functional near-infrared spectroscopy results reported by Rossi et al. [73] found differential responses to phonological legality in left fronto-temporal regions. This topographical result is at least consistent with our result showing that the P200 phonotactic probability effect was observed over left frontal areas only.

### Lexicality effects in N400 in the first presentation

The lexicality effect in N400 is consistent with numerous ERP studies [12], [13], [43], [48], [49]. This lexicality effect suggests differential processing to words compared to both pseudowords and nonwords with no difference in the N400 between pseudowords and nonwords. The failure to find a difference between pseudowords and nonwords is not predicted by lexical redintegration models. According to such models, after lexical access, more word neighbors should be activated by pseudowords than by nonwords. Since no differences between pseudowords and nonwords appeared in the N400 or any later epoch, it appears that, lexical redintegration did not occur in the initial presentation (i.e., during online auditory word recognition) as might have been predicted by lexically-based theories.

### Nonsignificant effects in the second presentation

Results for the second presentation were also inconsistent with lexical redintegration theories. According to these theories, nonwords should not receive any lexical-level redintegration during the second presentation, while pseudowords should be boosted by lexical redintegration from their word neighbors. However, there was no reliable difference between ERPs to pseudowords and nonwords in the second presentation. This negative finding was confirmed using a Bayes Analysis, which confirmed that the Bayes Factor for this effect was below the critical value of 1/3 [62].

An additional, though perhaps not very parsimonious, way to maintain support for lexical level representations would be to argue that, in the second presentation, top-down lexical processes occurred but were ‘cancelled out’ by bottom-up phonological processing effects. There is support for this stance; for example it has been argued, on the basis of computational simulations, that there are two causal routes through which vocabulary knowledge affects nonword repetition performance: either directly, or via effects on phonological memory functionality [67].

On the other hand, the lack of phonotactic probability effects in the second presentation are readily reconciled with lexical restructuring theory [66], [68] as follows. Following the initial presentation, phonological information from both pseudowords and nonwords is presented at equal strength to the lexicon. On the second presentation, sublexical fragments were equally active in each of these lexical conditions, and thus ERPs to pseudowords and nonwords did not differ in the present study.

It is also possible that the lack of significant ERP differences between pseudowords and nonwords indicates a problem in our paradigm. The second presentation was an immediate repetition. Given that even 4–5-year olds have an average memory span of 3 items [74], perhaps memory traces remained in working memory in a sufficiently intact form to render immediate repetitions of words, pseudowords and nonwords equivalent.

### Repetition effects

Although ERPs in the second presentation were equivalent across the 3 lexicalities, repetition effects in N400 between the first and second presentation were different for the 3 lexicalities, i.e., N400s to words showed reliable repetition effects across the three clusters, but N400s to pseudowords did not show repetition effects in the right temporoparietal and parietal clusters, nor did N400s to nonwords show repetition effects in the right temporoparietal cluster.

Our N400 repetition results look different from Deacon et al. [12]'s results. Although our study closely resembled this study in terms of stimulus production, the analysis is different. In the Deacon study, words, derived nonwords (corresponding to pseudowords in the present study) and nonderived nonwords (corresponding to nonwords in the present study) showed consistent repetition effects in N400, while in the present study, only words showed reliable repetition effects in N400. However, the repetition effects in Deacon et al.'s study were not net repetition effects for the same stimuli between two presentations, but effects compared between repeated stimuli and other unrepeated stimuli. This familiarity effect is similar to the general repetition effect for all stimuli in P200 in the present study in which participants judged whether the phonological information had been presented before.

For the unreliable N400 repetition effects to pseudowords and nonwords, it is possible that neither pseudowords nor nonwords have lexical representation and the immediate second presentation provided another opportunity to recheck the lexical status for these two distinct types of nonword stimuli. N400s to pseudowords and nonwords did not show reliable repetition effects because of the need for further ‘lexical’ processing in the immediate second presentation, whereas the reliable repetition effects to words occurred because words required no further processing in the immediate repetition. Different repetition effects to the 3 lexicalities may be consistent with Rugg and Nagy's finding (1987) that shallow processing of nonword stimuli causes differential effects of repetition in comparison with legal stimuli.

Our repetition effects in N400 can be explained by 2 opposing processes in nonword repetition priming [75], [76]. In a lexical decision task, performance for repeated nonwords is a net result of opposing processes: (1) a facilitatory effect of episodic memory retrieval for repeated nonwords; (2) and an inhibitory effect due to global familiarity. In the first presentation, nonwords are new and temporarily stored in working memory. In immediate repeated presentation, nonwords can be retrieved from working memory, leading to increased performance (quicker response times or higher accuracy rates). On the other hand, global familiarity renders nonwords more “word-like”, leading to decreased performance (slower response times or lower accuracy rates) in repeated presentation(s). In our study, repetition follows immediately after the first presentation. The facilitatory effect may have shown as a general repetition effect in P200, while the inhibitory effect may have shown as the unreliable repetition effects for pseudowords and nonwords in N400. This N400 in the immediate second presentation may have indexed a confirmatory lexical processing for more word-like nonwords which is at the same strength as the lexical processing in the first presentation.

## Conclusion

In the present study, we found an early difference between pseudowords and nonwords in P200 in an initial auditory presentation; no further reliable difference between pseudowords and nonwords was observed. These results do not support the view that lexical knowledge affects auditory word processing independently of sublexical processes. Our results instead suggest that long-term lexical knowledge has its effect via sublexical processing as suggested by lexical restructuring theory. Further, when phonological processing is required in immediate repetition, no pure effect of lexical redintegration can be observed. Our data support bottom-up over top-down theories of processing of the spoken word, and have consequences for theoretical approaches to the process of word learning.

NOTES: 1. In principle, participants should not produce any phonological neighbors from nonwords. However, given our instructions, participants inevitably produced suggestions for nonwords. These suggestions were very varied, so the nonwords can be considered as good stimuli.

2. These results were based on analyses on individual ERP components only, i.e., we found the phonotactic probability effects on P200, but not on N400, and the lexicality effects on N400, but not on P200. To confirm that the three lexicalities showed different effects in P200 and N400, we ran a 2×3×2×5 repeated measure ANOVA with Component (P200 and N400), Lexicality (words, pseudowords and nonwords), Hemisphere (left and right) and Cluster (frontal, anterotemporal, temporoparietal, parietal and occipital) for the first presentation according to suggestions in Nieuwenhuis et al. [77]. The following interactions involving Component and Lexicality were significant: Component × Lexicality, F(2, 46) = 6.38, p = .004, Component × Lexicality × Cluster, F(3.71, 85.22)  = 7.05, p<.001, respectively. Thus, words, pseudowords and nonwords showed different effects in P200 and N400.

## Supporting Information

Table S1 **Words, Pseudowords and Nonwords used in the study.**
(DOC)
