# Supplementary material for: Immediate Auditory Repetition of Words and Nonwords: An ERP Study of Lexical and Sublexical Processing
Source: PLoS One. 2014 Mar 18;9(3):e91988. doi: 10.1371/journal.pone.0091988 (PMC3958410; doi:10.1371/journal.pone.0091988)
Supplement: Table S1 — Words, Pseudowords and Nonwords used in the study. (DOC) [file pone.0091988.s001.doc]

**Supporting Information Table S1: Words, Pseudowords and Nonwords used in the study**

Words

| aunt | boot | cake | cart | ditch | dog | fork |
| --- | --- | --- | --- | --- | --- | --- |
| fruit | game | greed | height | hurt | lion | net |
| park | pet | pot | queen | rod | ship | smash |
| smile | soap | stove | tent | vase | wash | block |
| brush | burn | cheese | cone | dress | glove | grape |
| heat | hide | joke | lap | march | note | page |
| peach | pig | rain | shirt | snake | step | sting |
| swim |  |  |  |  |  |  |

Pseudowords

| ownt /ont/ | voot /vut/ | cabe /keb/ | cark /kak/ |
| --- | --- | --- | --- |
| vitch /vItʃ/ | deg /dɛg/ | forsh /fɔʃ/ | freet /frit/ |
| gome /gom/ | grood /grud/ | deight /dait/ | furt /fɜt/ |
| hion /haiɜn/ | zet /zɛt/ | pake /pek/ | thet /θɛt/ |
| fot /fɔt/ | wod /wɔd/ | shap /ʃæp/ | smap /smæp/ |
| smirle /smɜl/ | voap /vop/ | stuve /stuv/ | ggent /gɛnt/ |
| tase /taz/ | waish /waiʃ/ | bluck /blʌk/ | bruth /brʌð/ |
| burm /bɜm/ | cheem /tʃim/ | done /don/ | driss /drIs/ |
| glofe /glɔf/ | prape /prep/ | heash /hiʃ/ | houde /haud/ |
| goke /gok/ | thap /θæp/ | narch /natʃ/ | nurt /nɜt/ |
| tage /tedʒ/ | zeach /zitʃ/ | shig /ʃIg/ | nain /nen/ |
| tirt /tɜt/ | pnake /pnek/ | stek /stɛk/ | sphing /sfIŋ/ |
| swoum /swom/ |  |  |  |

Nonwords

| dwace /dwes/ | dwer /dwɜ/ | frerph /frɜf/ | geiched /dʒetʃt/ |
| --- | --- | --- | --- |
| ghlauff /glɔf/ | ghwie /gwai/ | ghwoute /gwaut/ | glaughc /glɔk/ |
| glowdge /glaudʒ/ | gwar /gwa/ | gwudd /gwʌd/ | kweuce /kwus/ |
| muippth /mupθ/ | olch /ɔltʃ/ | phrurch /frɜtʃ/ | quouth /kwauθ/ |
| rheimth /remθ/ | rooppth /rupθ/ | thweighp /θwep/ | thwir /θwɜ/ |
| ulv /ʌlv/ | usp /ʌsp/ | yeabb /jib/ | yieg /jaig/ |
| zighth /zait/ | cuithed /kuθt/ | dwar /dwa/ | ghlerf /glɜf/ |
| ghwir /gwɜ/ | gloughsh /glɔʃ/ | gnolge /nɔldʒ/ | gwawque /gwɔk/ |
| knarthe /nað/ | osp /ɔsp/ | thainge /θendʒ/ | thougnth /θaunθ/ |
| threrg /θrɜg/ | thruick /θruk/ | thwa /θwa/ | thwi /θwai/ |
| thwurmb /θwɜm/ | twawdge /twɔdʒ/ | ulch /ʌltʃ/ | veffth /vɛfθ/ |
| warthe /wað/ | yirlt /jɜlt/ | yolmn /jɔlm/ | zeiche /zeʃ/ |
| zighg /zaig/ |  |  |  |
